# Ipsilateral snare technique for the safe delivery of the Evolut PRO transcatheter aortic valve system: a case report

**DOI:** 10.1093/ehjcr/ytad555

**Published:** 2023-11-08

**Authors:** Motoki Fukutomi, Takayuki Onishi, Tomo Ando, Tetsuya Tobaru

**Affiliations:** Department of Cardiology, Kawasaki Saiwai Hospital, 31-27, Kawasaki, Kanagawa 212-0014, Japan; Department of Cardiology, Kawasaki Saiwai Hospital, 31-27, Kawasaki, Kanagawa 212-0014, Japan; Department of Cardiology, Kawasaki Saiwai Hospital, 31-27, Kawasaki, Kanagawa 212-0014, Japan; Department of Cardiology, Kawasaki Saiwai Hospital, 31-27, Kawasaki, Kanagawa 212-0014, Japan

**Keywords:** Ipsilateral snare technique, Transcatheter aortic valve implantation, Evolut transcatheter heart valve, Case report

## Abstract

**Background:**

Transcatheter aortic valve implantation (TAVI) requires several bail-out techniques for safe valve delivery and deployment. Particularly in cases of challenging aortic anatomy, the snare technique from the contralateral side of the surgical site can facilitate delivery of the transcatheter heart valve (THV) system. However, there are no previous reports of the snare technique from the ipsilateral side of the surgical site in TAVI cases.

**Case summary:**

A 77-year-old woman presented with severe aortic stenosis and congestive heart failure. As computed tomography showed a heavily calcified aortic arch, we performed TAVI using the ipsilateral snare technique to control the direction of the Evolut THV system. There was no haematoma or excessive bleeding at the surgical site during the procedure, and the patient was discharged without complications.

**Discussion:**

In cases with challenging anatomy of the aorta, the ipsilateral snare technique may be useful for the safe delivery of the Evolut system.

**Learning points**

- The snare technique was useful for the safe delivery of a self-expanding transcatheter heart valve system in a case with challenging aortic anatomy.
- Step-by-step procedures of ipsilateral snare technique for transcatheter aortic valve implantation.

## Introduction

Transcatheter aortic valve implantation (TAVI) has emerged as the standard treatment for elderly patients with severe aortic stenosis (AS). However, in cases with severe calcification at the outer curvature of the aortic arch, smooth and safe delivery of the transcatheter heart valve (THV) system can be difficult, especially with the Evolut heart valve system. The snare technique, usually performed from the contralateral part of the surgical site, is known as the one of the solutions to facilitate the delivery of the THV system through the challenging aortic anatomy. In this report, we present a case of successful TAVI using the ipsilateral snare technique for the delivery of an Evolut THV in a patient with a heavily calcified aortic arch.

## Summary figure

**Table:** 

12 July 2022	Hospital admission for congestive heart failure with severe AS.
	Left ventricular ejection fraction (LVEF) was 24%.
18 July 2022	Transcatheter aortic valve implantation with ipsilateral snare technique.
27 July 2022	Hospital discharge without any complications. Pre-discharge LVEF was 57%.
29 August 2022	Follow-up echocardiography showed a normal LVEF of 67%.
30 May 2023	The patient did not show any symptoms of heart failure.

## Case presentation

**Figure 1:**
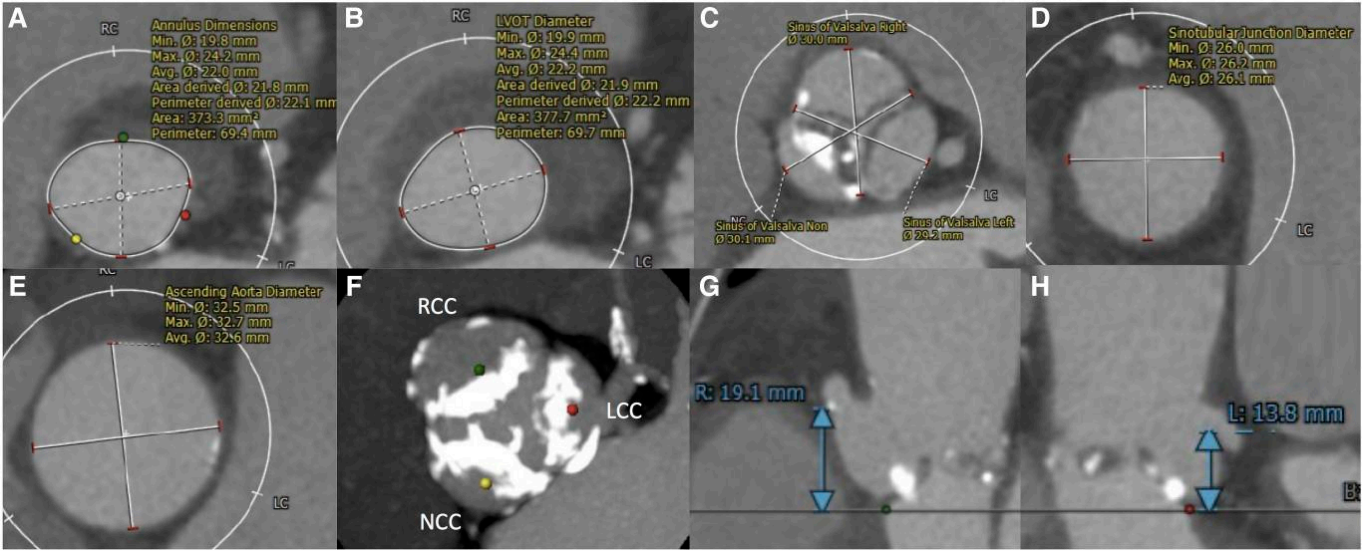
Computed tomography measurements before transcatheter aortic valve implantation. (*A*) The aortic annulus. (*B*) The left ventricular outflow tract. (*C*) The sinus of Valsalva. (*D*) The sinotubular junction. (*E*) The ascending aorta. (*F*) Leaflet calcification. (*G*) Right coronary artery height. (*H*) Left coronary artery height. LCC, left coronary cusp; NCC, non-coronary cusp; RCC, right coronary cusp.

**Figure 2:**
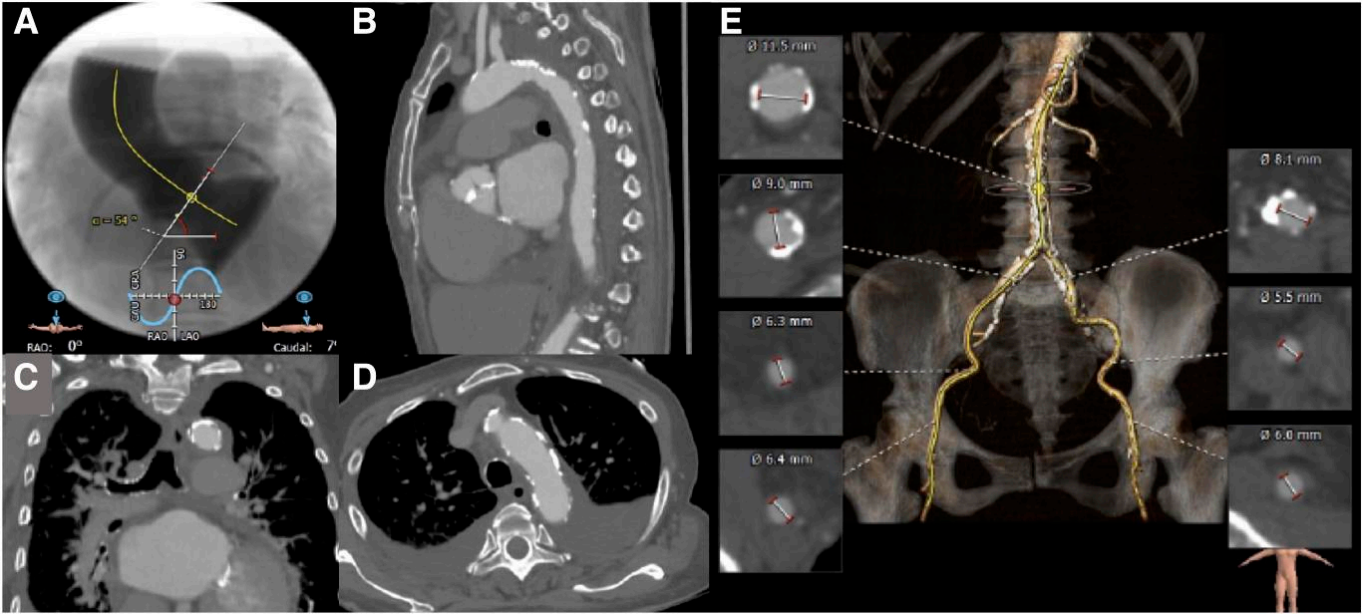
Computed tomography angiography images. (*A*) Aortic root angle. (*B*) Sagittal view of the aortic arch. (*C*) Coronal view of the aortic arch. (*D*) Axial view of the aortic arch. (*E*) Iliofemoral access route.

**Figure 3:**
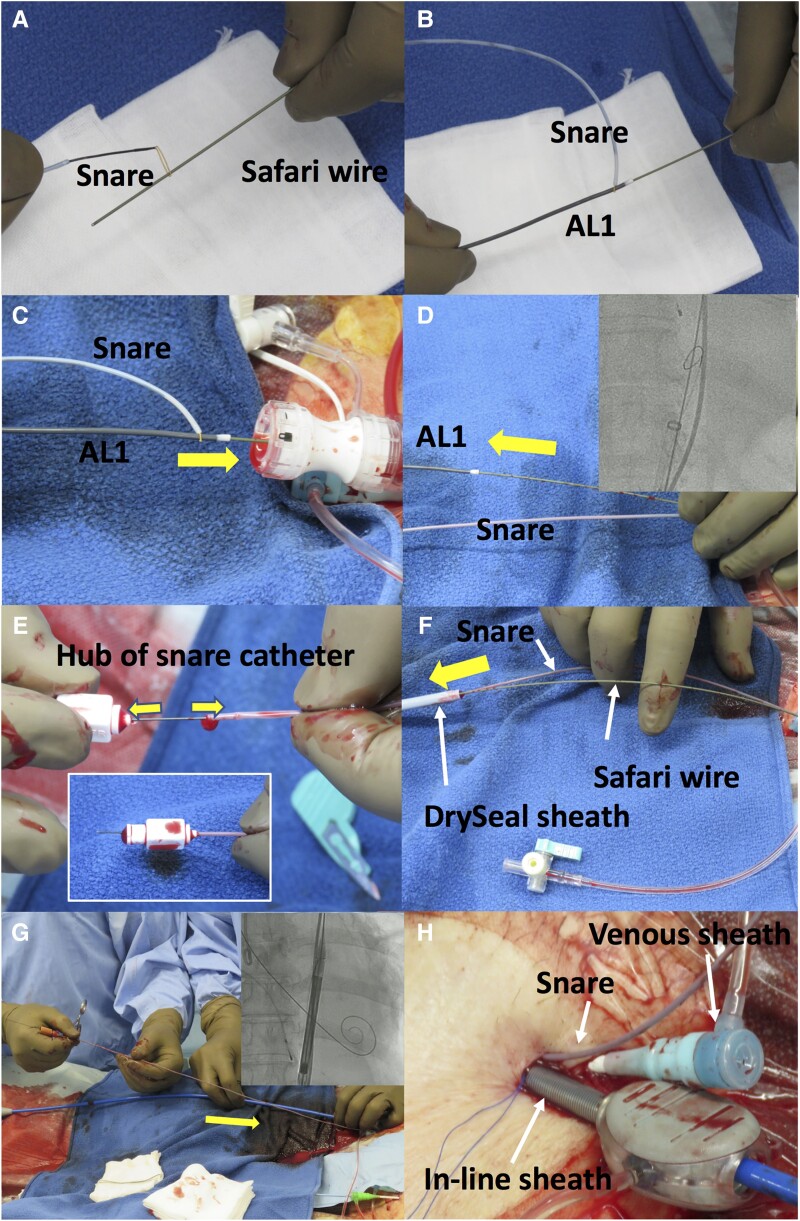
Step-by-step procedures of the ipsilateral snare technique. (*A*) A snare loop passed through the end of a Safari wire. (*B*) A 5-Fr AL1 catheter on a Safari wire was grasped with a snare. (*C*) The snare was then inserted into the DrySeal sheath together with the AL1. (*D*) AL1 was removed after the advancement of the snare. (*E*) The hub of the snare was cut off. (*F*) The DrySeal sheath was removed. (*G*, *H*) The Evolut system was inserted into the femoral artery just beside the snare using in-line sheath. The yellow arrows indicate the direction of motion of (C, D) AL1 catheter, (E) hub and snare cathter after cutting, (F) Dryseal sheath and (G) Evolut system.

**Figure 4:**
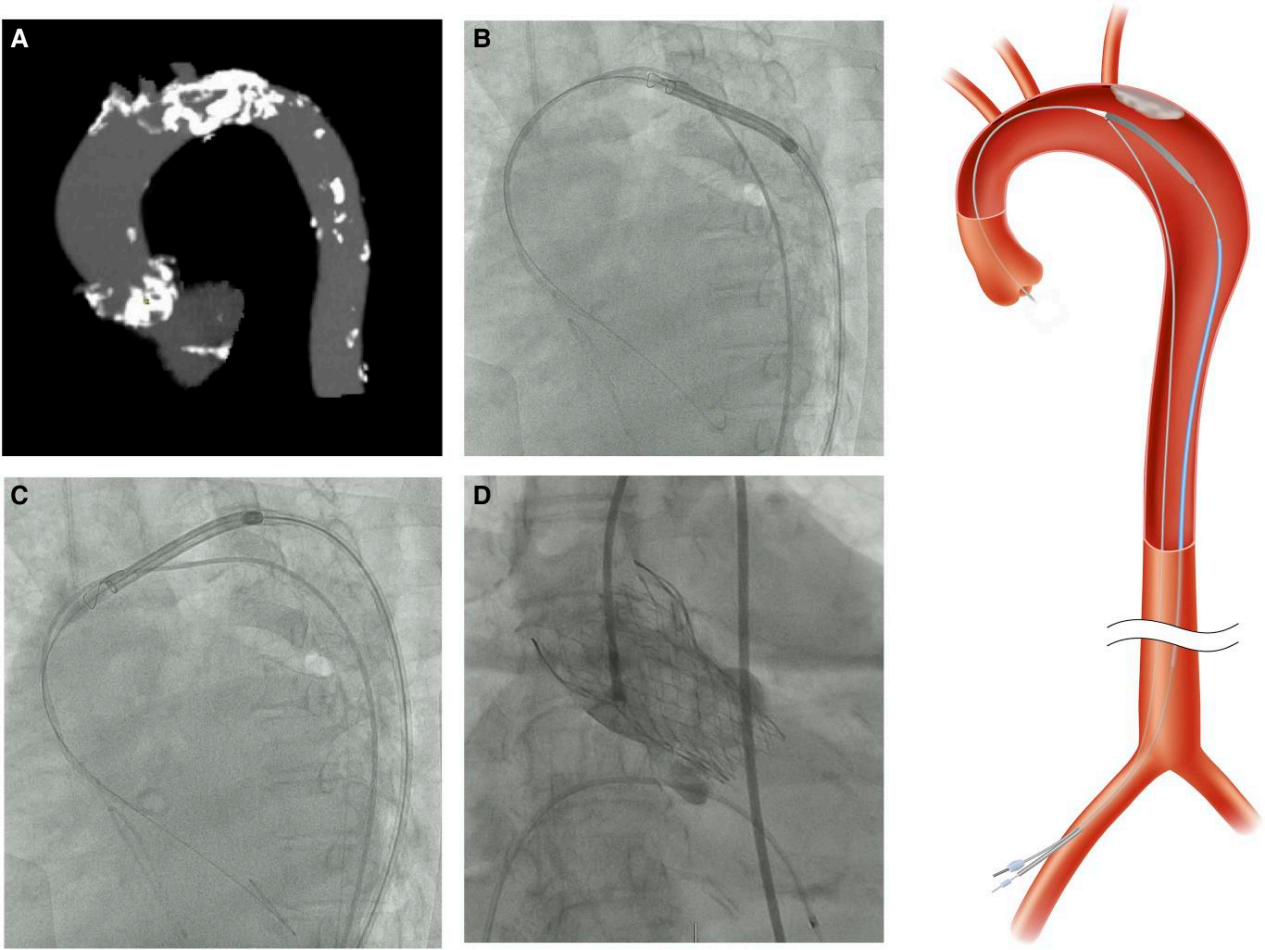
Ipsilateral snaring to control the direction of the Evolut system. (*A*) Heavy calcification of the aortic arch. (*B*, *C*) The Evolut system was successfully passed through the aortic arch without touching the calcification. (*D*) Successful valve deployment.

A 77-year-old woman with severe AS was admitted to our hospital with congestive heart failure. Laboratory tests showed a significant increase in brain natriuretic peptide of 2296 pg/mL without an increase in creatine kinase. Echocardiography showed very severe AS with a peak flow velocity of 6.4 m/s and a LVEF of 24%. Coronary computed tomography (CT) angiography showed no significant stenosis in the coronary arteries. Transcatheter aortic valve implantation was scheduled after receiving medical treatment for congestive heart failure. Based on CT findings (annulus perimeter 69.4 mm, bulky leaflet calcification), trans-femoral (TF) TAVI with an Evolut PRO+ 26 mm (Medtronic, Minneapolis, MN, USA) was planned (*Figure 1*). As CT revealed heavy calcification at the aortic arch (*Figure 2*), we planned to attempt ipsilateral snaring via a surgical TF access site (minimum diameter: 6.3 mm) to avoid touching this calcification. After placing the Safari wire (Boston Scientific, Marlborough, MA, USA) in the LV, the end of the wire was passed through the loop of a 10 mm Amplatz Goose Neck™ snare (Medtronic, Minneapolis, MN, USA; *Figure 3A*). To advance the snare catheter to the aorta smoothly, the tip of the AL1 catheter on the Safari wire was grasped with a snare outside the body (*Figure 3B*), and then both the AL1 and snare were delivered to the descending aorta through a14-Fr Gore DrySeal sheath (W.L. Gore and Associates, AZ, USA; *Figure 3C*). After removal of the AL1 (*Figure 3D*), we cut off the hub of the snare catheter with a small knife (*Figure 3E*) and removed the DrySeal sheath, leaving behind the snare catheter (*Figure 3F*). The Evolut THV was inserted into the same vessel immediately beside the snare catheter using an in-line sheath (*Figure 3G* and *H*). The nose cone of the THV was grasped by a snare at the descending aorta (*Figure 3G*), and the system was advanced to the aortic arch. The THV was successfully passed through the calcified aortic arch (*Figure 4A*) without interference from heavy calcification, using controlled traction of the nose cone by the snare (*Figure 4B* and *C*; Supplementary material online, *Videos S1* and *S2*). After releasing the snare, the THV was deployed safely (*Figure 4D*). Haemostasis at the surgical site was performed using a single Perclose Proglide™ (Abbott Vascular, USA). There was no excessive bleeding or haematoma at the surgical site during the procedure, and the patient was discharged without complications. Pre-discharge echocardiography showed that the LVEF had improved to 57%. The patient had no symptoms of heart failure in the 10 months after discharge.

## Discussion

Major vascular complications, including iatrogenic aortic dissection, are life threatening after TAVI.^1,2^ Iatrogenic aortic dissection may occur in cases involving a severely tortuous aorta or heavy calcification of the aortic arch. In these cases, a tortuous aorta or chunk of calcium often prevents smooth passage of the THV system, leading to injury of the aortic wall when the THV is forced through. The Edwards Sapien valve system incorporates a deflectable flex catheter, facilitating safe delivery of the THV device under all anatomical conditions. In contrast, the Medtronic Evolut valve system does not have this deflectable function. In our case, the patient had bulky leaflet calcification and reduced LVEF with congestive heart failure. We therefore chose a self-expanding Evolut valve to avoid annulus rupture and haemodynamic instability due to rapid pacing during valve deployment. The snare technique, which can directly control the direction of the Evolut THV system, may be a solution for TAVI cases with a severely tortuous or calcified aorta. This snare method can also be used as a bail-out technique to facilitate THV passage through the aortic valve in cases with a horizontal aorta or valve in valve.^3^

A typical snare procedure involves inserting a snare catheter via the contralateral femoral artery before crossing the aortic valve and then passing the catheter from the surgical site through the snare loop. After this catheter crosses the aortic valve, a Safari wire (or other LV support wires) is inserted into the LV. After these preparations, the THV system is advanced to the snare loop to be grasped. Therefore, if an LV support wire is already placed in the LV, it must be removed when performing this snare technique. However, the ipsilateral approach does not require this removal process, as shown in *Figure 1*, which is a major advantage. In our case, the ipsilateral snare worked well for the safe delivery and implantation of the THV without any vascular damages to the surgical site. This technique may be considered when an LV support wire is already present in the LV, or when the contralateral access site is highly stenosed or occluded. In our case, there was little bleeding from the surgical site, even when the in-line sheath and snare catheter were inserted into one hole of the vessel. However, if the procedure takes a long time, careful attention should be paid to bleeding from this site. Finally, this ipsilateral snare technique may also be useful for passing the THV through the heavily calcified bicuspid valve or horizontal aorta. In conclusion, the ipsilateral snare technique was useful for the safe delivery of the Evolut system by controlling the direction of the THV in cases with a challenging aortic anatomy.

## Supplementary Material

ytad555_Supplementary_Data

## Data Availability

The data underlying this article will be shared upon reasonable request to the corresponding author.

## Lead author biography

Motoki Fukutomi, MD, is a cardiologist, chief physician of Kawasaki Saiwai Hospital, Kawasaki, Japan. He graduated from Jichi Medical University in 2004. He finished his fellowship in structural heart intervention in Rigshospitalet, Copenhagen, Denmark (2018–20).

## Supplementary material

**Consent:** Written informed written consent for the publication of this case report, including images and related text, was obtained from the patient in accordance with the COPE guidelines.

**Funding:** None declared.
